# Tetracycline-Resistant Bacteria Selected from Water and Zebrafish after Antibiotic Exposure

**DOI:** 10.3390/ijerph18063218

**Published:** 2021-03-20

**Authors:** Ana Rita Almeida, Marta Tacão, Joana Soares, Inês Domingues, Isabel Henriques

**Affiliations:** 1 CESAM & Department of Biology, University of Aveiro, Campus Universitário de Santiago, 3810-193 Aveiro, Portugal; martat@ua.pt (M.T.); joana.soares2@ua.pt (J.S.); inesd@ua.pt (I.D.); 2 University of Coimbra, CESAM & Department of Life Sciences, Faculty of Science and Technology, Calçada Martins de Freitas, 3000-456 Coimbra, Portugal; ihenriques@ua.pt

**Keywords:** *Danio rerio*, pathogenicity test, multidrug resistance, qPCR, microcosm

## Abstract

The emergence of antibiotic-resistant pathogens due to worldwide antibiotic use is raising concern in several settings, including aquaculture. In this work, the selection of antibiotic-resistant bacteria (ARB) and antibiotic resistance genes (ARGs) was evaluated after exposure of zebrafish to oxytetracycline (OTC) for two months, followed by a recovery period. The selection of ARB in water and fish was determined using selective media. The abundance of *tetA* genes was estimated through qPCR. Higher prevalence of ARB was measured in all samples exposed to the antibiotic when compared to control samples, although statistical significance was only achieved five days after exposure. Isolates recovered from samples exposed to the antibiotic were affiliated with *Pseudomonas* and *Stenotrophomonas*. Various antibiotic susceptibility profiles were detected and 37% of the isolates displayed multidrug resistance (MDR). The selection of the *tetA* gene was confirmed by qPCR at the highest OTC concentration tested. Two MDR isolates, tested using zebrafish embryos, caused significant mortality, indicating a potential impact on fish health and survival. Overall, our work highlights the potential impact of antibiotic contamination in the selection of potential pathogenic ARB and ARGS.

## 1. Introduction

In the next decades, due to human population growth, the pressure on land and resources will increase. Thus, to meet food demand, intensive and semi-intensive systems for livestock and agriculture production are increasingly being used. For instance, in aquaculture, one of the fastest growing food sectors [1], fish are usually cultivated in overcrowded conditions, which increases stress and facilitates pathogen spread. Due to its low-cost production and broad-spectrum efficacy, oxytetracycline (OTC) is one of the most widely used antibiotics in the food production industry in Europe [2]. Since organisms cannot metabolize all the ingested antibiotic, the compound is excreted and dispersed into the aquatic environment [3]. Consequently, OTC has been detected not only in aquaculture wastewater [4,5] but also in rivers and surface water around the world [6,7].

The selective pressure that antibiotics exert on environmental bacteria, even at low concentrations, may lead to the selection of antibiotic-resistant bacteria (ARB) and to increased prevalence of antibiotic resistance genes (ARGs). Indeed, ARG abundance in aquatic and terrestrial ecosystems has been increasing since the 1940s [8,9]. For instance, ARG abundance has been found at concentrations ranging from 7.0 × 10^1^ to 5.9 × 10^6^ copies/mL in surface water and 4.2 × 10^2^ to 2 × 10^8^ copies/g in river sediments in China [10]. Moreover, values ranging from 3.7 × 10^7^ copies/mL to 9.1 × 10^7^ copies/mL have been found in aquaculture ponds contaminated with antibiotics [11]. Since ARGs can be transmitted to other bacteria through a variety of horizontal gene transfer strategies (e.g., via conjugation), they can be easily disseminated, especially in water systems [12,13], representing a health risk to humans and ecosystems. Therefore, ARGs have been indicated as environmental pollutants, and aquatic environments are considered large reservoirs of ARB and ARGs [9]. Several studies suggested the selection of ARB and ARGs following antibiotic exposure in aquatic environments. For instance, Seyfried et al. [14] showed a higher prevalence of tetracycline resistance genes (*tet*) in water from fish farms with recent OTC use; Harnisz et al. [6] showed that fish farms using OTC had an impact on the water of Drweca river by increasing the diversity of *tet* genes; and Huang et al. [11] revealed that the abundance of ARGs in fish culture ponds was higher than in control ponds. However, to our knowledge, the effect of OTC exposure in the selection of ARB and ARGs in a controlled environment (e.g., microcosms), which allows the reduction of confounding variables, has been rarely addressed [15,16].

The transfer of ARB and ARGs from the environment to humans or animals may promote the spread of antibiotic-resistant zoonotic pathogens. Indeed, some authors established a relationship between the use of antibiotics, namely tetracyclines, and the transfer of ARB and resistant pathogens to fish and humans (e.g., *Aeromonas*, Enterobacteriaceae and *Pseudomonas*) [17,18,19]. Per year, fish infections account for about 15% to 20% of production loss in aquaculture in China, posing great economic costs [20]. Pathogens such as *Aeromonas* spp., *Pseudomonas* spp. and several Enterobacteriaceae are frequently reported in aquaculture, causing disease in fish and other aquatic animals [20,21]. Hence, food-animals have been pointed out as reservoirs of ARGs and antibiotic-resistant pathogens [22], which can be transferred to humans through the food chain. Nevertheless, to our knowledge, most studies conducted in aquacultures have been directed at analyzing the bacterial communities in water, and therefore, the selection of ARB and ARGs within the organism’s microbiome is poorly understood.

In previous works, we have demonstrated that long-term exposure to OTC cannot only affect the organism itself (e.g., at the level of energy metabolism), but can also cause changes in the bacterial communities of fish and water [23,24]. In this work, we aim to study the effect of a long-term exposure to OTC on ARB and ARGs selection in zebrafish and water. We applied an innovative strategy, relying on exposure in microcosms, which allows conducting the experiment under controlled conditions, eliminating confounding variables. In addition, long-term exposure has rarely been applied in previous studies and is more realistic considering environmental contamination. Since up to 99% of environmental bacteria are uncultivable [25,26], we combined culture-dependent and culture-independent methods in order to obtain more comprehensive and reliable data.

## 2. Materials and Methods

### 2.1. Zebrafish Culture and Exposure

Zebrafish (*Danio rerio*) adults were obtained from the zebrafish culture established at the Biology Department of University of Aveiro (Aveiro, Portugal). The fish were kept under controlled conditions (temperature 27 °C, pH 7.5 ± 0.5, conductivity 800 ± 50 µS and dissolved oxygen ≥ 95%) in a recirculation system as described by Domingues et al. (2016) [27]. Zebrafish adults, AB strain with 4 months old, were selected for the experimental assays as recommended by OECD guideline 230 [28]. A total of 81 adults were exposed to two concentrations of oxytetracycline hydrochloride (0.01 and 10 µg/mL) for two months, via water, under semi-static conditions as described by Almeida et al. (2019b) [23]. Control animals were kept in identical conditions without antibiotic. Fish were randomly divided in aquaria (1 L): there were 27 fish in the control group (*n* = 9; 3 fish per aquarium within 3 replicates) and 54 fish in exposure groups (27 fish exposed to 0.01 µg/mL and 27 fish exposed to 10 µg/mL). After exposure, organisms were kept for five days in clean water (culture water) for recovery. The concentrations used in this experiment were selected based on our previous works where effects on fish and water microbiome were observed after exposure to 0.01 and 10 µg/mL OTC [23,24]. The lowest concentration tested (0.01 µg/mL) was found in aquaculture systems [5], while the highest concentration (10 µg/mL) was selected to understand the mechanisms of action of OTC in the exposure conditions and the effects of the antibiotic in a worst-case scenario. During the experiment, fish were fed daily with the commercial pellet Gemma Micro 500 food (Skretting^®^; Burgos, Spain), and water was renewed every three days to ensure water quality and OTC concentrations [23]. Samples were collected during the experiment at three different sampling moments: 5 days and 2 months of exposure (5 dE and 2 mE, respectively) and 5 days post-exposure (5 dPE). At each sampling point, samples from both water and fish were collected. To sample fish gut and skin bacteria, 9 fish per condition (two OTC concentrations plus the control) were euthanized with tricaine overdose (tricaine methane sulfonate, Metacain, MS-222; CAS number: 886–86–2) followed by spinal cord severing. Zebrafish fins were removed and placed in 3 mL of lysogeny broth (LB) medium and incubated at room temperature with smooth agitation until processing (Section 2.2.1); fish guts were aseptically removed and stored at −80 °C until analysis (Section 2.3.1). To select water bacteria through culture-dependent methods, water samples were immediately processed (Section 2.2.1). For culture-independent analysis, 100 mL of water was collected in triplicate and filtered using 0.22 mm hydrophilic PVDF durapore membrane filter (Merck Millipore; Massachusetts, EUA) for DNA purification (Section 2.3.1). Samples were then stored at −80 °C until further analysis.

### 2.2. Culture-Dependent Analyses

#### 2.2.1. Bacterial Counts and Strains Isolation and Identification

Zebrafish skin bacteria, preincubated in LB medium as described in the Section 2.1, were collected by filtrating 100 µL of medium through 0.45 µm pore membranes (Pall Corporation, Ann Arbor, MI, USA). To collect bacteria from water, 100 µL was filtrated through 0.45 µm pore membranes (Pall Corporation; Ann Arbor, MI, MA, USA). Bacteria retained in the membranes, from both fish skin and water, were then incubated for 24 h at 30 °C in the following media: Membrane Fecal Coliform agar (mFC), *Pseudomonas Aeromonas* Selective agar (Glutamate Starch Phenol Red Agar; GSP) and Plate Count Agar (PCA), all supplemented with 16 µg/mL of TET. After this period, colony forming units (CFUs) were quantified. The counts were determined from the samples exposed to the antibiotic and from the control (nonexposed) samples.

Tetracycline (TET)-resistant bacteria were selected from samples (fish skin and water) exposed to the highest concentration of OTC (10 µg/mL). Sixty antibiotic-resistant isolates from each culture media (30 isolates from fish skin and 30 isolates from water from GSP and mFC) were randomly selected, purified and stored for further analysis. Identification was based on the 16S rRNA gene as described by Silva et al. [26]. The PCR programs and primers are described in Table S1.

#### 2.2.2. Detection of Tetracycline Resistance and Integrase Encoding Genes

The occurrence of tetracycline resistance genes was inspected by PCR. Different target genes were selected, namely genes encoding efflux pumps (*tet*A, *tet*B, *tet*C, *tet*D, *tet*E and *tet*G) and ribosomal protection proteins (*tet*M and *tet*O). Additionally, the presence of integrase encoding genes *intI1* and *intI2* was analyzed. PCR mixtures (25 µL) were obtained by adding the DNA (1 µL of cell suspension) to nuclease-free water (16.25 µL), NZYTaq 2 × Green Master Mix (6.25 µL; 2.5 mM MgCl2; 200 mM dNTPs; 0.2 U/µL DNA polymerase) (NZYTech, Lisbon, Portugal) and the respective primers (0.75 µL of each in a 10 mM solution) (Table S1). The PCR primers and programs are described in Table S1.

#### 2.2.3. Antibiotic Susceptibility Testing

The isolates’ antibiotic susceptibility profile was determined using the disk diffusion method on Mueller-Hinton Agar (MH) according to the European Committee on Antimicrobial Susceptibility Testing (EUCAST) guidelines [29,30]. The selection of antibiotic and disk concentrations was based on the EUCAST recommendations for the genera of the isolates. The antibiotics tested were the following: aztreonam (ATM; 30 mg), cefepime (FEP; 30 mg), ceftazidime (CAZ; 10 mg), chloramphenicol (C; 30 mg), ciprofloxacin (CIP; 5 mg), gentamicin (CN; 10 mg), imipenem (IMI; 10 mg), ticarcillin (TIC; 75 mg), ticarcillin/clavulanic acid (TIM; 75 mg TIC + 10 mg clavulanic acid), tigecycline (TGC; 15 mg) and trimethoprim/sulfamethoxazole (STX; 25 mg) (Oxoid, Basingstoke, UK). The reference strain used for quality control was the *Escherichia coli* ATCC 25922. Plates were incubated at 30 °C for 18 h and the inhibition zone diameters were measured. Isolates were then classified as “susceptible, standard dose” (S), “susceptible, increased exposure” (I) or “resistant” (R) according to EUCAST expert rules and CLSI breakpoint tables for the gentamicin [30,31].

#### 2.2.4. Zebrafish Pathogenicity Test

To assess strain pathogenicity, we selected potential fish pathogenic bacteria based on the phylogenetic affiliation (i.e., *Stenotrophomonas maltophilia*) and also included isolates resistant to four classes of antibiotic (isolates M-W6, M-W12, M-W16, M-W18, M-S9, M-S13 and M-S14; Table S2). Selected strains were grown in LB overnight at 30 °C with agitation. The optical density (OD) of each liquid culture (in LB medium) was measured at 600 nm in a spectrophotometer UV mini-1240 (UV-VIS Spectrometer, Shimadzu). Bacterial concentrations were adjusted to 10^8^ CFU/mL in sterile fish system water [32].

On the day before mating, zebrafish adults (AB strain) were placed in rearing aquaria and left for mating. Zebrafish eggs were collected within 30 min after natural mating and rinsed in fish system water. Then, embryos were screened using a stereomicroscope (Stereoscopic Zoom Microscope-SMZ 1500, Nikon Corporation, Tokyo, Japan) to exclude unfertilized or injured embryos.

Embryos 24 h postfertilization (hpf) were manually dechorionized for exposure, and pools of 20 organisms in triplicate were used for exposure to each test strain. Embryos were then exposed by static immersion for 5 h to each bacterial strain (10^8^ CFUs/mL) in a petri dish as described by Milligan-Myhre et al. [32]. After this period, organisms were removed and washed twice in sterile fish system water. Then, embryos were transferred to new petri dishes and kept in water from the fish system in a temperature-controlled room. Organisms were observed daily under a stereomicroscope for a 4-day period. Mortality was recorded and dead organisms were removed daily. The evaluation of development alterations was not considered. However, no deformations were observed during the test period.

### 2.3. Culture-Independent Analyses

#### 2.3.1. Environmental DNA Extraction

Total DNA extraction of zebrafish gut and water was performed as described by Almeida et al. [33]. Briefly, zebrafish gut DNA was extracted using the PowerSoil^®^ DNA isolation kit (MOBIO laboratories, Carlsbad, CA, USA) following the manufacturer’s instruction. Water DNA extraction was performed using the commercial kit Genomic DNA Purification kit (Thermo Fisher Scientific; Waltham, MA, USA) as described by Henriques et al. [34].

#### 2.3.2. Quantitative Polymerase Chain Reaction (qPCR)

The abundance of total bacteria (using the 16S rRNA gene as a proxy) and the abundance of the *tet*A resistance gene were determined by qPCR using the primer sets 338F/518R and tetAF/tetAR, respectively (Table S3). The PCR mixture and the temperature profile (Table S3) were as described by Silva et al. [35]. The melting curves for both genes were obtained from 55 to 95 °C, with steady 0.1 °C increments every 5 s. Standard curves were obtained by 10-fold dilutions in ultrapure water of plasmid DNA holding inserts of the target genes as described by Tavares et al. [36]. For each sample, the copy numbers of *tet*A resistance gene were normalized to the 16S rRNA gene copy numbers.

### 2.4. Statistical Analysis

Sigma plot V.12.5 (SysStat software Inc., San Jose, CA, USA) was used to calculate statistical differences due to OTC exposure. A *t*-test was used to calculate OTC effects in the selection of TET-resistant bacteria at each sampling time. The one-way ANOVA test was used to calculate differences in *tet*A gene abundance (qPCR) between samples exposed to OTC and control samples, and to assess the significance of the pathogenic effect of our isolates. The Shapiro-Wilk test was used to test normality. A Kruskal-Wallis test followed by the post hoc Dunnett’s test was used when the normality test failed. A significance level of 0.05 was considered.

## 3. Results and Discussion

### 3.1. Effects of OTC in the Abundance of Tetracycline-Resistant Bacteria

**Figure 1:**
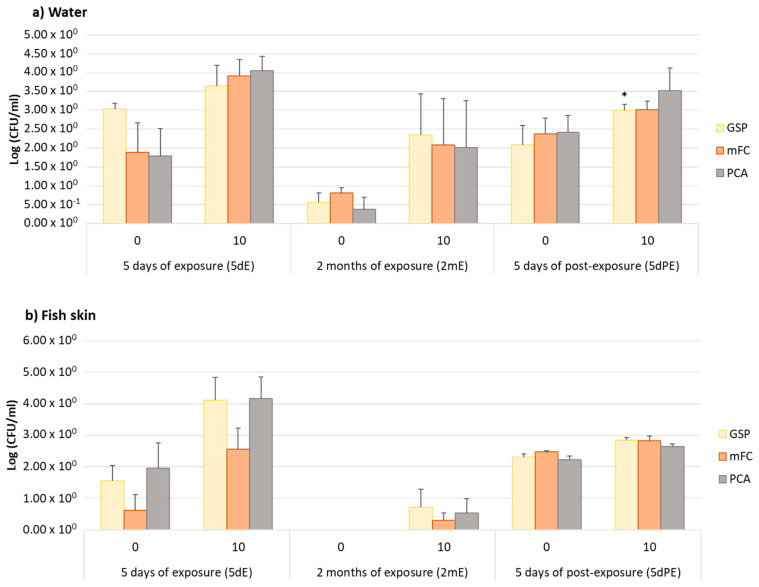
Total counts (Log (CFU/mL)) of antibiotic-resistant bacteria from nonexposed (0 µg/L) and oxytetracycline (OTC)-exposed (10 µg/L) samples at each sampling time (5 days of exposure: 5 dE; 2 months of exposure: 2 mE and 5 days of postexposure: 5 dPE). Counts were determined in selective media (Glutamate Starch Phenol Red Agar (GSP), Membrane Fecal Coliform agar (mFC) and Plate Count Agar (PCA)) supplemented with tetracycline (TET) at 16 µg/mL. Asterisks (*) represent statistically significant differences (*p* ≤ 0.05) towards the respective control (nonexposed water and fish skin).

The total counts of tetracycline (TET)-resistant bacteria, expressed in Log (CFU/mL), are represented in Figure 1. Selective media were chosen to cultivate potential antibiotic-resistant fish pathogens (GSP medium) and human pathogens (mFC medium). The abundance of tetracycline-resistant bacteria was higher in both water and fish skin exposed to OTC towards control samples (nonexposed water and fish skin), regardless of the sampling time and culture medium. However, due to the variability observed between replicates, this difference was only statistically significant for water samples at 5 dPE in GSP medium (*p* = 0.05). Our experimental design, namely the use of individual aquaria (each aquarium with 3 fish represents a replicate) in a semi-static condition, may explain the high variability among replicates. After exposure ceased, the total counts of resistant bacteria increased. It is known that OTC has a bacteriostatic effect [37]. Therefore, the increase of antibiotic-resistant bacteria (ARB) after the exposure ceased was expected. Evidence of the selection of ARB due to antibiotic use was observed in aquaculture environments [6,38]. Nevertheless, in in situ studies, several factors may influence these results, which may represent a limitation. In our study, it was possible to eliminate environmental variables and still observe the selection effect.

### 3.2. Taxonomic Affiliation of Isolates

To determine the taxonomic affiliation of ARB obtained from samples exposed to OTC, 60 isolates (30 isolates from fish skin and 30 isolates from water) from each culture medium (GSP and mFC) were identified. Results indicate that 37% of the isolates selected from water samples were identified as *Stenotrophomonas* (16 isolates in GSP medium and 6 isolates in mFC medium) and 63% as *Pseudomonas* (14 isolates in GSP and 24 isolates in mFC). Isolates selected from the fish skin were affiliated with *Stenotrophomonas* (35%; 17 isolates in GSP and 4 isolates in mFC) and *Pseudomonas* (65%; 13 isolates in GSP and 26 isolates in mFC). These genera present intrinsic characteristics that provide resistance against tetracyclines. For instance, *Pseudomonas* have low membrane permeability, express efflux pumps that may expel toxic compounds and are also able to produce antibiotic-inactivating enzymes [39]. *Stenotrophomonas*, on the other hand, express multi-drug efflux pumps and have the capacity to form biofilms [40], which are known to provide additional resistance to antibiotics [41]. In addition, both *Stenotrophomonas* and *Pseudomonas* have the capacity to biodegrade tetracyclines, allowing these genera to use this antibiotic as a carbon source and to survive in its presence [19,42].

*Stenotrophomonas* and *Pseudomonas* are considered pathogenic to fish and humans. Although these genera are naturally present in healthy fish, the abundance of both *Stenotrophomonas* and *Pseudomonas* can significantly increase in diseased fish skin [43]. For instance, some *Stenotrophomonas* species are known to cause enteritis and cutaneous hemorrhages in fish [44]. Moreover, *Stenotrophomonas* spp. may also cause septicemia and pneumonia in humans [45].

Since we did not analyze isolates obtained from samples not exposed to OTC, we cannot exclude the presence in those samples of bacteria with characteristics identical to those described here. However, considering the CFU counts, the prevalence of these bacteria will be higher after exposure to the antibiotic.

### 3.3. Antibiotic Resistance Profile and Resistance Genes

**Figure 2:**
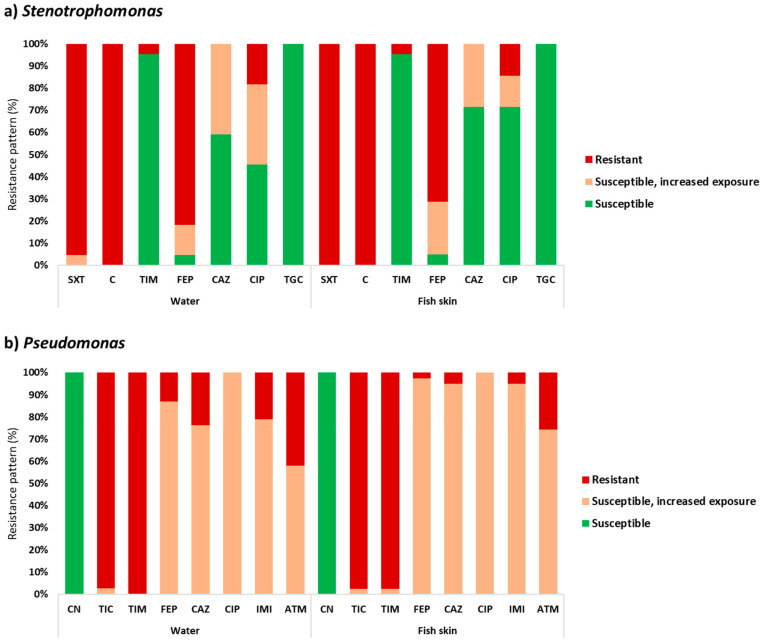
Resistance pattern (susceptible; susceptible, increased exposure; and resistant) of isolated bacteria according to the genera (*Stenotrophomonas* and *Pseudomonas*) and type of sample (water or fish skin). Aztreonam: ATM; cefepime: FEP; ceftazidime: CAZ; chloramphenicol: C; ciprofloxacin: CIP; gentamicin: CN; imipenem: IMI; ticarcillin: TIC; ticarcillin/clavulanic acid: TIM; tigecycline: TGC; and trimethoprim/sulfamethoxazole: STX.

Antibiotic resistance profiles of the selected isolates are represented in Figure 2. *Stenotrophomonas* isolates were resistant to chloramphenicol (100% in water and fish skin) and trimethoprim/sulfamethoxazole (water: 95% and fish skin: 100%) followed by cefepime (water: 82% and fish skin: 71%). All *Stenotrophomonas* isolates were revealed to be sensitive to tigecycline. Regarding *Pseudomonas*, isolates were resistant to ticarcillin/clavulanic acid (water: 100% and fish skin: 97%) and ticarcillin (97% in water and fish skin). All *Pseudomonas* isolates were shown to be sensitive to gentamicin and displayed a reduced susceptibility to ciprofloxacin. These results suggest that the resistance profiles differ according to the origin (water or fish skin). In fact, bacterial communities of both matrices are influenced by distinct factors. For instance, fish mucus is a complex fluid containing several proteases, lectins and antimicrobial peptides, which acts a barrier against pathogens and harbors a highly specific bacterial community [46,47,48]. Indeed, a previous work reported multidrug resistance among bacteria from freshwater fish mucus [49].

The isolation of strains resistant to three or more classes of antibiotics (multidrug resistant) was also verified (Table S2). *Stenotrophomonas* included a higher number of multidrug-resistant isolates (water: 82% and fish skin: 76%) compared to *Pseudomonas* (water: 21% and fish skin: 5%). Notably, the resistance profile of our isolates included resistance to antibiotics used in clinical settings to treat human infections. For instance, trimethoprim/sulfamethoxazole is usually the primary choice to treat *Stenotrophomonas* infections in humans [50,51]. Nevertheless, in the last years, the prevalence of *Stenotrophomonas* resistant to this antibiotic has been increasing [51]. In accordance, in our work, 97% to 100% of our isolates were resistant to trimethoprim/sulfamethoxazole.

Alternatives to this antibiotic have been suggested, such as tigecycline and moxifloxacin, which have shown to be highly effective against *Stenotrophomonas* in in vitro tests [52,53]. Regarding *Pseudomonas*, antibiotics like colistin, aminoglycosides and ceftolozane/tazobactam are usually prescribed for clinical treatment [54,55]. From the antibiotics tested in this work, only tigecycline and gentamicin were effective against 100% of the *Stenotrophomonas* and *Pseudomonas* isolates, respectively. Therefore, this result indicates that OTC exposure may coselect for bacteria resistant to other antibiotics.

It is known that bacteria may acquire antibiotic resistance mechanisms through vertical or horizontal gene transfer. In fact, in literature, it was reported that some tetracycline resistance genes like *tet*B, *tet*C, *tet*E, *tet*S, *tet*O and *tet*M are usually associated to transferable elements [56,57,58]. In our work, the presence of *tet* resistance genes was inspected by PCR; however, no *tet* genes were detected in any of the isolates. Moreover, the search for integrases (*intI*1 and *intI*2) was also carried out. These elements are usually associated with mobile ARGs [59]. Nevertheless, in our work, no integrase genes were found. The use of selective media supplemented with 16 µg/mL (minimal inhibitory concentration: MIC for *Enterobacteriaceae*) [31] of TET might be high enough to prevent bacteria from harboring *tet* resistance genes to grow, promoting the growth of intrinsically resistant bacteria [60]. Additionally, another hypothesis may be related with the fact that the proportion of ARB harboring *tet* genes may be so small that the increasing prevalence of intrinsically resistant bacteria may mask their presence [60]. Therefore, the absence of acquired *tet* and *intI* genes suggests a predominance of intrinsically resistant bacteria.

### 3.4. Pathogenicity Test

**Figure 3:**
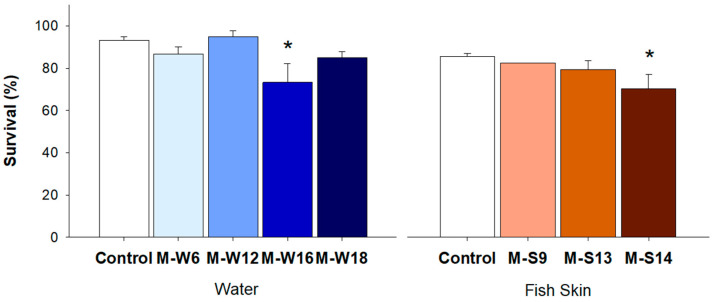
Survival percentage (%) of zebrafish embryos exposed to multidrug-resistant bacteria isolated from water and zebrafish skin. Strains were obtained from samples exposed to 10 µg/mL of OTC, after a 5-day postexposure period (5 dPE) period. Asterisks (*) represent statistically significant differences (*p* ≤ 0.05) towards the control.

Zebrafish embryos were used to evaluate the pathogenicity of seven multidrug resistant *Stenotrophomonas* strains. In fact, zebrafish embryos present several advantages like optical transparency and translational potential to humans, allowing a powerful tool to study host–pathogen interactions [61,62]. Overall, all the strains induced mortality in zebrafish embryos (Figure 3), although differences relative to the control were not statistically significant for five strains. Only two isolates revealed significant results, namely one isolate from water (M-W16; *p* = 0.004) and one isolate from fish skin (M-S14; *p* = 0.018). The infection via immersion tests the capacity of a pathogen to cross the fish’s natural barrier (e.g., fish skin), simulating the natural exposure pathway [63]. Nonetheless, it is not possible to determine the exact number of bacteria that can invade the organism [63]. Furthermore, the inclusion of additional controls, including previously described pathogenic and nonpathogenic strains, would have been important to further validate the results obtained. However, the results are an indicator that our isolates have a pathogenic effect and can cause mortality in fish. Therefore, future studies applying other endpoints like fish immunological response or the strains’ virulence factors may allow us to obtain a deeper understanding of the pathogenic potential of these isolates.

### 3.5. Abundance of tetA gene in Water and Fish Gut

**Figure 4:**
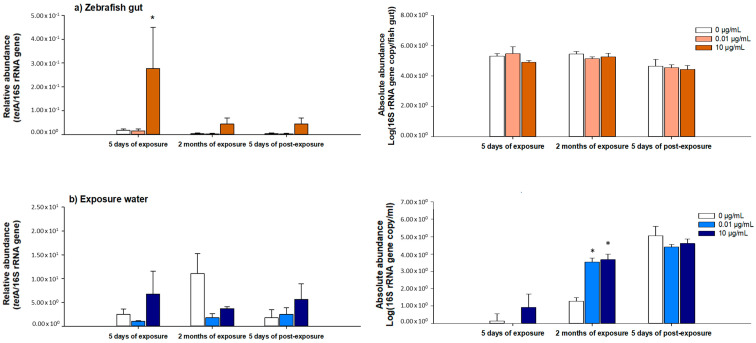
Relative abundance of *tet*(A) and absolute abundance of 16S rRNA gene (Log gene copy number/mL or fish gut), determined by qPCR in (**a**) zebrafish gut and (**b**) water exposed to OTC (0, 0.01 and 10 µg/mL) at three sampling times (5 days of exposure: 5 dE; 2 months of exposure: 2 mE; and 5 days postexposure: 5dPE). Asterisks (*) represent statistically significant differences (*p* ≤ 0.05) towards the respective control (0 µg/mL).

The abundance of the tetracycline resistance gene *tet*A was assessed in zebrafish gut and water in three sampling times, namely during exposure at 5 dE and 2 mE and after exposure ceases at 5 dPE (Figure 4). Results were compared to control samples (nonexposed to the antibiotic). The 16S rRNA gene was quantified to estimate the absolute abundance of bacteria and to estimate the relative abundance of the *tet*A gene (*tet*A/16 S rRNA gene copy number). The qPCR reaction efficiency and correlation coefficients (R^2^) for both genes, 16S rRNA gene and *tet*A are presented in Table S4. One of the most frequently detected tetracycline resistance gene is *tet*A. Furthermore, this gene was already pointed as a potential indicator of tetracycline resistance gene abundance [11]. In our work, despite its low abundance, it was possible to detect the *tet*A gene in both zebrafish gut and water samples, irrespective of antibiotic exposure. Regarding the zebrafish gut, the relative abundance of *tet*A was higher in samples exposed to 10 µg/mL of OTC comparatively to the control, irrespective of sampling time. However, a statistically significant result (*p* = 0.05) was only observed for the first sampling moment (5 dE). On the other hand, no statistically significant differences were observed between OTC-exposed and control samples in bacteria abundance (16S rRNA gene absolute abundance), irrespective of the sampling moment. Concerning water, at 10 µg/mL of OTC, the same tendency was observed (i.e., higher prevalence of the *tet*A gene in comparison to control samples) for all sampling times except 2 mE, although differences were not statistically significant. Moreover, at 2 mE, there was an observed significant increase of bacteria abundance (16S rRNA gene absolute abundance increase; *p* ≤ 0.001) in OTC-exposed samples. The decrease in *tet*A abundance and the increase in bacterial abundance, observed at this sampling moment, suggest the selection of bacteria expressing other, and probably intrinsic, resistance mechanisms.

Given this, our results indicate that OTC may select ARB and promote the increase of ARG abundance, namely the *tet*A, although this increase is exposure time dependent. In fact, previous works reported that low concentrations such as 1 to 15 µg/L of TET may increase the abundance of resistance genes [60,64]. Moreover, it was also reported that the selection of *tet* resistance genes, also observed in this work, may also allow the coselection of ARGs to other antibiotics [60,65]. For instance, it was demonstrated that the coselection of *tet*A with *sul2* and *bla_TEM-1_* is possible due to the location of these genes in the same mobile genetic elements (e.g., plasmids) [65].

Hence, the use of culture-independent methods might be a useful methodology to complement the culture-dependent methods. This integrated approach may allow a better understanding of the antibiotic role in the selection of ARB and ARGs.

## 4. Conclusions

Our results indicate that the selection of TET-resistant bacteria from fish skin and water occurred. Even though significant results were only observed in water samples at 5 dPE in GSP medium, the total counts of tetracycline-resistant bacteria were consistently higher in OTC-exposed samples than control (nonexposed water and fish skin). The prevalence of *Pseudomonas* and *Stenotrophomonas* may be related with the expression of intrinsic resistance mechanisms. Regarding ARGs, no *tet* or integrase encoding genes were detected in our isolates. Analysis of susceptibility to other antibiotics revealed the occurrence of multidrug-resistant (MDR) isolates in samples exposed to the antibiotic. In addition, the pathogenicity test revealed that these MDR bacteria may induce mortality in zebrafish embryos. The *tetA* gene was enriched in samples exposed to the highest antibiotic concentration (10 µg/mL).

This study emphasizes the impact of antibiotics on the aquatic environment, particularly their role in the selection of potential antibiotic-resistant pathogens.

## Data Availability

Not applicable.

## Supplementary Materials

The following are available online at https://www.mdpi.com/1660-4601/18/6/3218/s1, Table S1: PCR primers and conditions of 16S rRNA gene, tetracycline resistance genes (tet) and integrases genes (int). Table S2: Resistance profile of multidrug resistant bacteria according to its medium selection (GSP: G or mFC: M medium) and type of sample (water: W or fish skin: S). Aztreonam: ATM; cefepime: FEP; ceftazidime: CAZ; chloramphenicol: C; ciprofloxacin: CIP; imipenem: IMI; ticarcillin: TIC; ticarcillin/clavulanic acid: TIM; trimethoprim/sulfamethoxazole: STX. Table S3: qPCR primers and conditions of 16S rRNA gene and tetracycline resistance genes (tet). Table S4: The qPCR reaction efficiency (E) and correlation coefficients (R2) for 16S rRNA gene and tetA at each sampling time (5 days of exposure: 5dE; 2 months of exposure: 2mE and 5 days of post-exposure: 5dPE).
