# Supplementary material for: Tetracycline-Resistant Bacteria Selected from Water and Zebrafish after Antibiotic Exposure
Source: Int J Environ Res Public Health. 2021 Mar 20;18(6):3218. doi: 10.3390/ijerph18063218 (PMC8003806; doi:10.3390/ijerph18063218)
Supplement: Supplementary file 1 [file ijerph-18-03218-s001.pdf]

Supplementary material

Tetracycline-resistant bacteria selected from water and zebrafish after antibiotic exposure

Ana Rita Almeida[1], Marta Tacão[1], Joana Soares[1], Inês Domingues[1] and Isabel Henriques[2]

1 - Department of Biology & CESAM, University of Aveiro, Campus Universitário de Santiago, 3810-193 Aveiro, Portugal

2 – University of Coimbra, CESAM & Department of Life Sciences, Faculty of Science and Technology, Calçada Martins de Freitas 3000-456 Coimbra, Portugal

Table S1: PCR primers and conditions of 16S rRNA gene, tetracycline resistance genes (*tet*) and integrases genes (*int*).

Target	Primer sequence	Amplicon Size	Annealing temperature (ºC)	Program	Reference
**16S rRNA**	27_F: AGAGTTTGATCCTGGCTCAG<br>1492_R: GGCTACCTTGTTACGACTT	1467	52	94 ºC - 3 min (1x)<br>94 ºC - 1 min<br>52 ºC - 1 min<br>72 ºC - 2 min<br>(30x)<br>72 ºC–10 min (1x)	Lane (1991)
***tet*(A)**	tetA_F: GCTACATCCTGCTTGCCTTC<br>tetA_R: GCATAGATCGCCGTGAAGAG	211	53	94 º C – 5 min (1x)<br>94 ºC – 30 s,<br>53 ºC – 30 s,<br>72 ºC– 30 s,<br>(35x)<br>72 ºC–7 min (1x)	Nawaz et al. (2006)
***tet*(B)**	tetB_F: TCATTGCCGATACCACCTCAG<br>tetB_R: CCAACCATCATGCTATTCCATCC	391			
***tet*(C)**	tetC_F: CTGCTCGCTTCGCTACTTG<br>tetC_R: GCCTACAATCCATGCCAACC	897			
***tet*(D)**	tetD_F: TGTGCTGTGGATGTTGTATCTC	844			

	tetD_R: CAGTGCCGTGCCAATCAG				
***tet*(E)**	tetE_F: ATGAACCGCACTGTGATGATG<br>tetE_R: ACCGACCATTACGCCATCC	744			
***tet*(G)**	tetG_F: GCGCTNTATGCGTTGATGCA<br>tetG_R: ATGCCAACACCCCCGGCG	803	55	94 ºC – 5 min (1x)<br>94 ºC – 30 s,<br>55 ºC – 30 s,<br>72 ºC– 30 s,<br>(35x)<br>72 ºC–7 min (1x)	Ng et al. (2001)
***tet*(M)**	tetM_F: GTGGACAAAGGTACAACGAG<br>tetM_R: CGGTAAAGTTCGTCACACAC	406			
***tet*(O)**	tetO_F: AACTTAGGCATTCTGGCTCAC<br>tetO_R: TCCCACTGTTCCATATCGTCA	515			
***tet*(S)**	tetS_F: CATAGACAAGCCGTTGACC<br>tetS_R: ATGTTTTTGGAACGCCAGAG	667			
intI1	intI1_F: CCTCCCGCACGATGATC<br>intI1_R: TCCACGCATCGTCAGGC	280	55	94 ºC – 5 min (1x)<br>94 ºC – 30 s,<br>55 ºC – 30 s,<br>72 ºC– 30 s,<br>(30x)<br>72 ºC–7 min (1x)	Kraft et al. (1986)
intI2	intI2_F: CCTCCCGCACGATGATC<br>intI2_R: TCCACGCATCGTCAGGC	233	50	94 ºC – 5 min (1x)<br>94 ºC – 30 s,<br>50 ºC – 30 s,<br>72 ºC– 30 s,<br>(30x)<br>72 ºC–7 min (1x)	Goldstein et al. (2001)

Table S2: Resistance profile of multidrug resistant bacteria according its medium selection (GSP: G or mFC: M medium) and type of sample (water: W or fish skin: S). Aztreonam: ATM; cefepime: FEP; ceftazidime: CAZ; chloramphenicol: C; ciprofloxacin: CIP; imipenem: IMI; ticarcillin: TIC; ticarcillin/clavulanic acid: TIM; trimethoprim/sulfamethoxazole: STX.

Strain	Closest relative strain	Accession number	Antibiotic resistance phenotype
G-W1	*Stenotrophomonas maltophilia* strain APP36	MT533812.1	FEP; C; SXT
G-W5	*Stenotrophomonas maltophilia* strain APP36	MT533812.1	FEP; C; SXT
G-W6	*Stenotrophomonas maltophilia* strain APP36	MT533812.1	FEP; C; SXT

G-W8	*Stenotrophomonas maltophilia* strain CGKV/J16a-2013	MK078536.1	FEP; C; SXT
G-W9	*Stenotrophomonas sp.* ROi7	EF219038.1	FEP; C; SXT
G-W10	*Stenotrophomonas maltophilia* strain APP36	MT533812.1	FEP; C; SXT
G-W13	*Stenotrophomonas maltophilia* strain B.xNS12	MT199173.1	FEP; C; SXT
G-W15	*Stenotrophomonas maltophilia* strain APP36	MT533812.1	FEP; C; SXT
G-W16	*Stenotrophomonas maltophilia* strain APP36	MT533812.1	FEP; C; SXT
G-W17	*Stenotrophomonas maltophilia* strain APP36	MT533812.1	FEP; C; SXT
G-W18	*Stenotrophomonas maltophilia* strain APP36	MT533812.1	FEP; C; SXT
G-W19	*Stenotrophomonas maltophilia* strain APP36	MT533812.1	FEP; C; SXT
G-W25	*Stenotrophomonas pavanii* strain S1-5	MT645772.1	FEP; C; SXT
G-S4	*Stenotrophomonas maltophilia* strain APP36	MT533812.1	FEP; C; SXT
G-S5	*Stenotrophomonas maltophilia* strain APP36	MT533812.1	FEP; C; SXT
G-S8	*Stenotrophomonas maltophilia* strain APP36	MT533812.1	FEP; C; SXT
G-S21	*Stenotrophomonas maltophilia* strain APP36	MT533812.1	FEP; C; SXT
G-S22	*Stenotrophomonas maltophilia* strain Ai 4	MN880434.1	FEP; C; SXT
G-S23	*Stenotrophomonas maltophilia* strain Ai 4	MN880434.1	FEP; C; SXT
G-S24	*Stenotrophomonas maltophilia* strain APP36	MT533812.1	FEP; C; SXT
G-S25	*Stenotrophomonas maltophilia* strain APP36	MT533812.1	FEP; C; SXT
G-S26	*Stenotrophomonas maltophilia* strain APP36	MT533812.1	FEP; C; SXT
G-S27	*Stenotrophomonas maltophilia* strain APP36	MT533812.1	FEP; C; SXT
G-S28	*Stenotrophomonas sp.* NA06056	CP054931.1	FEP; C; SXT
G-S30	*Stenotrophomonas maltophilia* strain APP36	MT533812.1	FEP; C; SXT
G-W14	*Pseudomonas alcaligenes* strain clon40	MN371833.1	TIC; TIM; CAZ; IMI
G-S12	*Pseudomonas sp.* strain Isyb05	KY678894.1	TIC; TIM; CAZ; IMI
G-S16	*Pseudomonas sp.* strain Atecer7E	MT386171.1	TIC; TIM; CAZ; IMI
M-W6	*Stenotrophomonas maltophilia* strain APP36	MT533812.1	TIM; C; SXT; CIP
M-W12	*Stenotrophomonas maltophilia* strain APP36	MT533812.1	FEP; C; SXT; CIP
M-W16	*Stenotrophomonas maltophilia* strain APP36	MT533812.1	FEP; C; SXT; CIP
M-W18	*Stenotrophomonas maltophilia* strain APP36	MT533812.1	FEP; C; SXT; CIP
M-S9	*Stenotrophomonas maltophilia* strain APP36	MT533812.1	FEP; C; SXT; CIP
M-S13	*Stenotrophomonas maltophilia* strain APP36	MT533812.1	FEP; C; SXT; CIP
M-S14	*Stenotrophomonas maltophilia* strain APP36	MT533812.1	FEP; C; SXT; CIP
M-W13	*Pseudomonas sp.* Pc102	LC420219.1	TIC; TIM; FEP; CAZ; IMI

M-W14	*Pseudomonas sp.* Pc102	LC420219.1	TIC; TIM; FEP; CAZ; IMI; ATM
M-W15	*Pseudomonas alcaligenes* strain clon40	MN371833.1	TIC; TIM; FEP; CAZ; IMI; ATM
M-W19	*Pseudomonas sp.* Pc102	LC420219.1	TIC; TIM; FEP; CAZ; IMI; ATM
M-W27	*Pseudomonas sp.* Pc102	LC420219.1	TIC; TIM; FEP; CAZ; IMI
M-W28	*Pseudomonas sp.* Pc102	LC420219.1	TIC; TIM; FEP; CAZ; IMI; ATM
M-W29	*Pseudomonas sp.* Pc102	LC420219.1	TIC; TIM; CAZ; IMI

Table S3: qPCR primers and conditions of 16S rRNA gene and tetracycline resistance genes (*tet*).

Target	Primer sequence	Program	Reference
**16S rRNA gene**	338F_ACTCCTACGGGAGGCAGCAG<br>518R_ ATTACCGCGGCTGCTGG	95 ºC - 3 min (1x)<br>95 ºC - 15 sec<br>65 ºC - 30 sec<br>(30x)	Muyzer et al. (1993)
***tet*(A)**	tetA_F: GCTACATCCTGCTTGCCTTC<br>tetA_R: GCATAGATCGCCGTGAAGAG	95 ºC - 10 min (1x)<br>95 ºC - 15 sec<br>60 ºC - 30 sec<br>(35x)	Nawaz et al. (2006)

Table S4: The qPCR reaction efficiency (E) and correlation coefficients (R^2) for 16S rRNA gene and *tet*A at each sampling time (5 days of exposure: 5dE; 2 months of exposure: 2mE and 5 days of post-exposure: 5dPE).

	**16S rRNA gene**		***tet*A**	
	**R^2**	**E (%)**	**R^2**	**E (%)**
**5 days of exposure (5dE)**	0.948	185.1	0.980	130.4
**2 months of exposure (2mE)**	0.979	146.4	0.993	103.8

**5 days of post-exposure (5dPE)**	0.975	171.0	0.974	88.8

References

Goldstein C, Lee MD, Sanchez S, et al (2001) Incidence of class 1 and 2 integrases in clinical and commensal bacteria from livestock, companion animals, and exotics. Antimicrob Agents Chemother 45:723–726. doi: 10.1128/AAC.45.3.723-726.2001

Kraft CA, Timbury MC, Platt DJ (1986) Distribution and genetic location of Tn7 in trimethoprim-resistant *Escherichia coli*. J Med Microbiol 22:125–131. doi: 10.1099/00222615-22-2-125

Lane DJ (1991) 16S/23S rRNA sequencing. In: E. Stackebrandt and M. Goodfellow (ed) Nucleic Acid Techniques in Bacterial Systematics. John Wiley & Sons, New York, pp 115–175

Muyzer G, De Waal E, Uitterlinden A (1993) Profiling of complex microbial populations by denaturing gradient gel electrophoresis analysis of polymerase chain reaction-amplified genes coding for 16S rRNA. Appl Env Microb 59:695–700. doi: 0099-2240/93/030695-06$02.00/0

Nawaz M, Sung K, Khan SA, et al (2006) Biochemical and molecular characterization of tetracycline-resistant *Aeromonas veronii* isolates from catfish. Appl Environ Microbiol 72:6461–6466. doi: 10.1128/AEM.00271-06

Ng LK, Martin I, Alfa M, Mulvey M (2001) Multiplex PCR for the detection of tetracycline resistant genes. Mol Cell Probes 15:209–215. doi: 10.1006/mcpr.2001.0363
